# Important Meeting at Leeds: Large Funds Forthcoming

**Published:** 1917-01-27

**Authors:** 


### THE ROYAL BRITISH COLLEGE OF NURSING.

### Important Meeting at Leeds: Large Funds Forthcoming.

(From the *Yorkshire Post* of January 20.)

A meeting to further the project of the College of Nursing was held on the afternoon of January 19 at the Philosophical Hall, Leeds. Alderman Charles Lupton, Chairman of the Leeds Infirmary, presided.

Alderman Lupton said when things got into their proper perspective we should see that one of the most striking changes which had occurred in the latter part of the nineteenth century was the great change which had arisen with regard to the nursing profession. It had always been the privilege of women from time immemorial to play the part of nurses to the sick, but the trained nurse we had to-day was a growth of the last fifty years. The beginning of trained nurses in England might be taken back to the time when Miss Nightingale carried out her heroic crusade at the time of the Crimean War, and it was largely owing to her impetus and the ambitions which she fostered as to the future of nursing that we had the modern trained nurse. If they looked at the record of the war they would find that the services of the nurses, coupled with the magnificent work which the physicians and surgeons had done, would have done almost as much to the winning of the war as the armies in the field. They had saved an extraordinary number of lives and had also been the means of preventing a great amount of personal distress. This progress could not have been effected without great sacrifice on the part of the nurses, and to-day, when they were hoping to make a further step forward, it was fitting that they should recall that one of the people who would be always remembered in connection with the war as a true heroine was a nurse—Miss Edith Cavell.

There was no general hall-mark to show the competent person, and each had to depend on the recommendation of a doctor or possibly the recommendation of the matron of a hospital. This was unfair to the properly trained nurse, and still more unfair to the patient, who might easily be entrusted to the care of a nurse who was absolutely unqualified. The only remedy was to have some society which should only admit nurses into its ranks who were really qualified to be members of the nursing profession, and that they should try to induce all nurses who had reached that level to join the society. In Yorkshire they hoped that in future no nurse would be admitted who had not reached that level; that some differentiation would be arranged in the certificates given to the nurses; and that precautions would be taken to ensure that the value of the training-school certificates should be retained, and that nurses should not obtain their certificates by proficiency in knowledge which could be obtained from books alone.

### The Proper Status of Nursing.

The Hon. Arthur Stanley, Chairman of the Council of the College, announced that on Thursday, at a largely attended meeting in London of the Royal British Nurses' Association, specially called for the purpose, that Association unanimously and formally ratified a deed of amalgamation with the College, under which the College dropped the word "Limited" from its title, and, subject to the approval of the Privy Council, would become the Royal British College of Nursing. This was a tremendous stride forward, and it was a privilege that they should be able to take their stand, in however humble a way, with the Royal College of Surgeons and the Royal College of Physicians. It had been objected that there were laymen on the Council of the College, but he reminded the meeting that the original Council remained in office only for two years, and at the end of that time every member of the College would have a vote in the election of the new Council.

### The Way to the Money.

The College could not do good work unless it had a satisfactory endowment. There were two great organisations which had collected very large sums of money for the Red Cross. One was an Association of ladies who had collected well on to £200,000 for the Home of the Paralysed at the Star and Garter. The other was a great organisation, which had raised £500,000. It was a hopeful sign that both of these organisations wanted to raise money for the purpose of the College of Nursing. He proposed to get them together to see whether their efforts could not be united to secure the College an endowment of which it might be proud. On taking up that work he soon discovered that the nursing profession was practically solid in favour of State Registration, and therefore that was an important plank in the platform. The College would be a great protection for the nurses in many ways. With regard to the question of a registered uniform he said it was not for a mere man, and especially a layman, to say anything about uniform that ladies were going to wear. In the Red Cross, during the last two years, they had probably had more changes in uniform than the British Army during the last fifty. He did not know whether a registered uniform would be a good thing or not, but he felt certain that a properly protected badge would be beneficial.

(*Continued on page* 350.)

### The Royal British College of Nursing Meeting at Leeds *(continued from p. 34?)*

MISS SWIFT ON THE COLLEGE.

Miss SWIFT said the College would be a democratic institution. After a period of two years the government would be by vote and direct representation, in the hands of the nurses themselves It was very important to the nursing profession that there should be uniformity of qualification secured by uniformity of curriculum, and a single portal examination. At present qualified nurses had no legal status, and that was why they wanted every nurse to join the College to make effective the demand for State Registration of trained nurses. For the period of grace, the first two years they were accepting nurses who had had a two years' training, but afterwards nurses who had not had a three years' training would not be recognised as trained nurses.

THE TRAINING OF NURSES.

A question was asked as to whether two or three years' experience in a military hospital was likely to be considered adequate training for a nurse.

Miss SWIFT said that training in a military hospital did not count. There were a great many helpers in this war, V.A.D.s and others, without whom the work of the war could not be carried on, but their experience could not count to them in the future. That made the present sacrifice greater. A military hospital was a special hospital. Answering another question, Miss Swift said they had three Poor-Law matrons and one member trained in a Poor-Law institution on the Council.

Mr. STANLEY said the Council had been in close touch with the Poor-Law Association and the Poor-Law Officers' Association.

Answering another question, Miss SWIFT said there would be a supplementary register of nurses who had devoted themselves to work amongst children.

AT THE HEAD OF THEIR PROFESSION.

Sir BERKELEY MOYNIHAN proposed a vote of thanks to Mr. Stanley and Miss Swift. When they first heard of this scheme, he said, they viewed the matter with grave anxiety and deep concern. The medical profession of this country realised that whatever might be said of the international standard of its physicians and surgeons, there was only one opinion in the whole world with regard to the international standard of our nurses. They were always placed at the head of their profession all over the world, and the medical profession's anxiety and apprehension were rather that something might be done insidiously to lower the standard of training of nurses in this country. It was at first rumoured that there might be a scheme afoot by which a very short period of training would enable a nurse to get her name on the Register. It was satisfactory to him to know that the minimum period of training would be three years at least. Nurses must necessarily be of different grades, and Sir Berkeley suggested that the nurses with the highest qualifications and endowments might be recognised as "Fellows" of the College of Nursing.

Lieutenant-Colonel LITTLEWOOD seconded the vote of thanks, and suggested that Mr. Stanley might well be made one of the first "Fellows" of the College of Nursing. The vote was heartily accorded, and a similar compliment was paid to the Chairman.

